# Evaluation of skin prick location on the forearm using a novel skin prick automated test device

**DOI:** 10.3389/falgy.2023.1289031

**Published:** 2023-11-01

**Authors:** Sven F. Seys, Senne Gorris, Saartje Uyttebroek, Wout Backaert, Mark Jorissen, Rik Schrijvers, Rembert Daems, Dirk Loeckx, Laura Van Gerven, Peter W. Hellings

**Affiliations:** ^1^ Research Department, Hippo Dx, Aarschot, Belgium; ^2^ Department of Otorhinolaryngology-Head and Neck Surgery, UZ Leuven, Leuven, Belgium; ^3^ Laboratory of Experimental Otorhinolaryngology, Department of Neurosciences, KU Leuven, Leuven, Belgium; ^4^ Allergy and Clinical Immunology Research Group, Department of Microbiology, Immunology & Transplantation, KU Leuven, Leuven, Belgium; ^5^ Department of General Internal Medicine, UZ Leuven, Leuven, Belgium; ^6^ Department of Otorhinolaryngology, Academic Medical Center, University of Amsterdam, Amsterdam, Netherlands; ^7^ Department of Otorhinolaryngology, University of Ghent, Ghent, Belgium

**Keywords:** skin prick test, allergy diagnosis, standardization, histamine, wheal

## Abstract

**Background:**

The skin prick test (SPT) is the gold standard for identifying allergic sensitization in individuals suspected of having an inhalant allergy. Recently, it was demonstrated that SPT using a novel skin prick automated test (SPAT) device showed increased reproducibility and tolerability compared to the conventional SPT, among other benefits.

**Objective:**

This study aimed to evaluate prick location bias using the novel SPAT device.

**Methods:**

A total of 118 volunteers were enrolled in this study and underwent SPATs with histamine (nine pricks) and glycerol control (one prick) solutions on the volar side of their forearms. Imaging of the skin reactions was performed using the SPAT device, and the physician determined the longest wheal diameter by visually inspecting the images using a web interface. Prick location bias was assessed along the medial vs. lateral and proximal vs. distal axes of the forearm.

**Results:**

In total, 944 histamine pricks were analyzed. Four medial and four lateral histamine pricks were grouped, and wheal sizes were compared. The longest wheal diameters were not significantly different between the medial and lateral prick locations (*p* = 0.41). Furthermore, the pricks were grouped by two based on their position on the proximal–distal axis of the forearm. No significant difference was observed among the four groups of analyzed prick locations (*p* = 0.73).

**Conclusion:**

The prick location on the volar side of the forearm did not influence wheal size in SPAT-pricked individuals.

## Introduction

The skin prick test (SPT) and serum-specific IgE test are both commonly used to evaluate type I hypersensitivity in patients with suspected inhalant allergy (1). Previous studies indicated that SPT is a more sensitive diagnostic test than the serum-specific IgE test (2, 3). Recently, it has been shown that screening for sensitization to allergens using *in vitro* molecular tests exhibits lower sensitivity than the extract-based SPT (4).

Despite the clear clinical need for the SPT and the availability of guideline recommendations (1, 5, 6), a considerable variation in its application persists due to both operator- and device-dependent factors (7, 8). To address this challenge, a skin prick automated test (SPAT), which is a novel device to automate and standardize the SPT procedure, has been developed. The device performs the prick procedure and the imaging of the prick reaction. A previous study by Gorris et al. (9) showed that the SPAT is associated with reduced intrasubject variability and patient discomfort while maintaining high levels of sensitivity and specificity.

According to international guidelines, the SPT is performed on the volar side of the forearm or the back of the patient (1, 6). However, previous literature is inconclusive on whether prick location on different positions of the forearm impacts the outcome of the test result (10, 11). In addition, these studies were performed in rather small datasets. Therefore, we evaluated prick location bias using the SPAT device, eliminating other variables such as prick force, device, or operator.

## Methods

### Study design

The setup of the previous SPAT validation study (9), which involved nine pricks of histamine (10 mg/ml) as positive controls and one prick of glycerol-saline solution as a negative control, allowed us to address our research question. The study was approved by the institutional review board of UZ Leuven (S66403) and registered online at www.isrctn.com (ISRCTN14098475).

### Recruitment

All study participants provided written informed consent before inclusion in the study. Healthy volunteers, irrespective of their atopic status, between 18 and 65 years old were eligible for the study.

**Figure 1:**
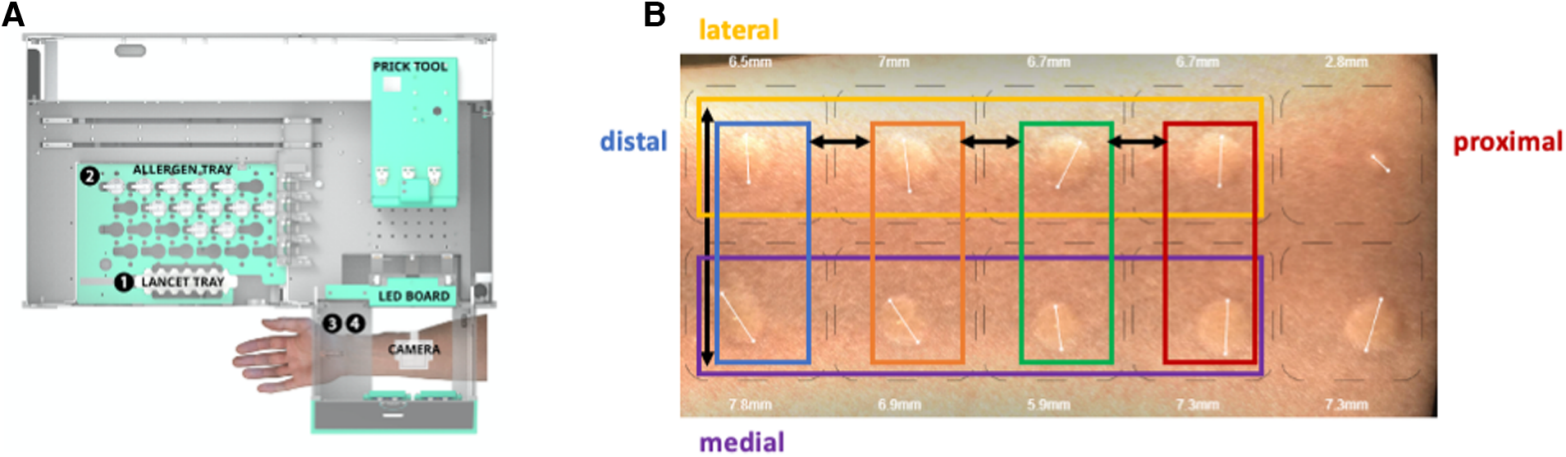
Study setup. (**A**) Inside view of the SPAT medical device with the prick tool moving first to position 1, the lancet tray, to collect the lancets, then moving to position 2, the allergen tray, to collect the allergens from the vials, and finally moving to position 3, where the arm is positioned for the prick procedure. After 15 min, the arm is positioned at position 4, where the camera is also located, taking 35 images of the volar side of the arm. (**B**) Representative image of the skin reaction 15 min after a skin prick automated test (SPAT). The prick locations on the volar side of the arm are indicated.

The prick procedure was performed using the SPAT medical device (Hippo Dx, Aarschot, Belgium; Figure 1A). In brief, the study participants positioned their forearms against the foreseen location of the SPAT device after the operator started the testing procedure on the touch screen and the automated pricking procedure was started. A total of 10 pricks were performed simultaneously by the device on the volar side of the forearm.

After 15 min, the SPAT device was utilized to conduct imaging of the skin reaction, and the physician analyzed the images of the skin reactions in a web interface to determine the longest wheal diameter.

### Statistics

The Shapiro–Wilk test was used to evaluate normality. The Mann–Whitney or Kruskal–Wallis test was employed to conduct between-group comparisons of nonparametric data. A *p*-value of <0.05 was considered statistically significant.

## Results

Data were collected from 118 volunteers, with each individual undergoing eight pricks (four rows on the proximal–distal axis and two rows on the medial–lateral axis) (Figure 1B). Prick location bias was assessed along the medial vs. lateral axis and proximal vs. distal axis on the volar side of the forearm. The histamine pricks were categorized into groups of two or four depending on their position along the proximal–distal or medial–lateral axis of the forearm, respectively (Figure 1A).

**Figure 2:**
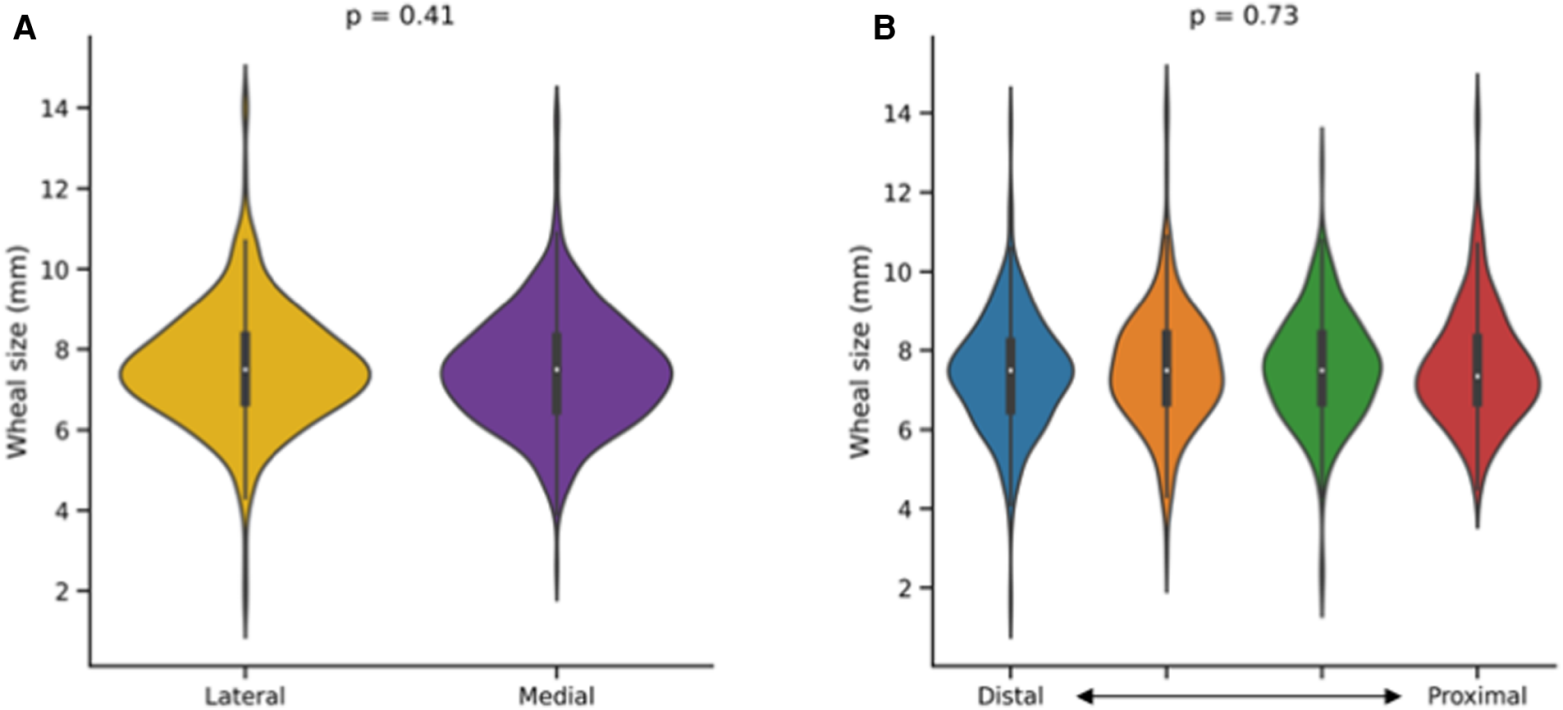
Impact of prick location on the outcome of a skin prick automated test (SPAT). Wheal sizes (longest wheal diameter) were compared along the lateral–medial (**A**) and proximal–distal (**B**) axes of the forearm. Mann–Whitney and Kruskal–Wallis tests were used for between-group comparisons.

In total, 944 histamine pricks were analyzed. The longest wheal diameters were not significantly different between medial [median with interquartile range: 7.5 mm (6.5–8.3)] and lateral [7.5 mm (6.7–8.3)] prick locations (*p* = 0.41, Figure 2A). Furthermore, considering the proximal–distal axis, no significant difference was observed among the four groups [7.4 mm (6.7–8.3), 7.5 mm (6.7–8.4), 7.5 mm (6.7–8.4), 7.5 mm (6.5–8.2)] of prick locations analyzed (*p* = 0.73, Figure 2B).

## Discussion

In the present study, we showed that the prick location on the volar side of the forearm did not influence the wheal size in SPAT-pricked individuals. Previous studies predominantly consist of older studies that have shown inconsistent results. In their study, Swain and Becker (10) conducted intradermal testing utilizing similar doses of histamine and observed a significant increase in wheal sizes in proximity to the elbow compared with the wrist. Several other studies have demonstrated differences in skin sensitivity to allergens on the arm, exhibiting a similar pattern with the cubital fossa being more reactive than the sites near the wrist (12–15).

These reports differ from the findings of Clarke et al. (11), who investigated skin reactions to common aeroallergens in 35 individuals with asthma using conventional skin prick testing and did not observe any significant influence of the prick location on the patient's forearm. Furthermore, the study conducted by Demoly et al. (16) could not detect a significant difference in skin reactivity to histamine when comparing the medial and lateral sides of the forearm in a sample of eight healthy volunteers. Since these studies were performed in small datasets or several decades ago, we argue that it is not possible to draw any firm conclusions.

In addition, it should be noted that exogenous histamine induces wheals directly by binding to histamine 1 and 4 receptors, whereas allergens first require binding to allergen-specific IgE and subsequent mast cell degranulation to release endogenous histamine (17). Although the expected skin reactions may therefore be different, the use of exogenous histamine allows easy evaluation of intrasubject variations of skin reactions.

Our current study is more robust compared to the previous reports in several aspects. Most importantly, performing a skin test using the SPAT device excludes the influence of operator-dependent factors such as prick force and human errors, often leading to false-positive or false-negative results (8). Second, the number of tested individuals and thus the number of pricks being analyzed are approximately 3–4 times higher. Third, our study was performed with a concentration of 10 mg/ml of histamine, which is currently widely used as a positive control in skin tests.

In conclusion, we are convinced that the use of an automated SPT for skin testing, as demonstrated in this study with the SPAT device, will play a significant role in establishing a standardized approach to allergy testing in future clinical practice.

## Data Availability

The data analyzed in this study is subject to the following licenses/restrictions: The dataset is not published online. Requests to access these datasets should be directed to the corresponding author.
